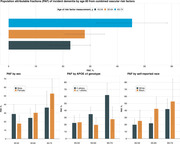# The combined impact of midlife and late‐life vascular risk factors on 33‐year incident dementia: the Atherosclerosis Risk in Communities Study

**DOI:** 10.1002/alz.084043

**Published:** 2025-01-09

**Authors:** Jason R Smith, James R. Pike, Elizabeth Selvin, Pamela L. Lutsey, Priya Palta, David S. Knopman, B Gwen Windham, Josef Coresh, Richey Sharrett, Alden L. Gross, Jennifer A. Deal

**Affiliations:** ^1^ Johns Hopkins University, Baltimore, MD USA; ^2^ Johns Hopkins Bloomberg School of Public Health, Baltimore, MD USA; ^3^ University of Minnesota School of Public Health, Minneapolis, MN USA; ^4^ University of North Carolina Chapel Hill, Chapel Hill, NC USA; ^5^ Mayo Clinic, Rochester, MN USA; ^6^ University of Mississippi Medical Center, Jackson, MS USA; ^7^ Johns Hopkins University Bloomberg School of Public Health, Baltimore, MD USA

## Abstract

**Background:**

Midlife vascular risk factors are associated with an increased risk of dementia. However, the overall contribution of modifiable vascular risk factors in midlife and late‐life to dementia remains unclear. In this study, we quantified population attributable fractions, which account for risk factor prevalence and strength of relative risks, of incident dementia from vascular risk factors measured in midlife and early late‐life.

**Method:**

We used 33 years of prospective cohort data in the Atherosclerosis Risk in Communities Study. Incident dementia (community surveillance and algorithmic diagnosis with expert clinical adjudication) was ascertained from baseline (1987‐1989) through December 31, 2020. We quantified population attributable fractions of incident dementia by age 80 from the combination of hypertension (systolic blood pressure ≥130, diastolic blood pressure ≥80, or use of medication), diabetes (fasting glucose ≥126, non‐fasting glucose ≥200, self‐reported physician diagnosis of diabetes, or use of medication for diabetes/insulin), and self‐reported current cigarette smoking (at least one risk factor versus none) measured at different ages (45‐54 [n = 7,731], 55‐64 [n = 12,273]; 65‐74 [n = 6,660]). We also computed attributable fractions stratified by sex, apolipoprotein ε4 genotype, and self‐reported race.

**Result:**

The prevalence of having one or more of the vascular risk factors was 61.6% (95% CI: 60.5, 62.7%) at age 45‐54 and increased to 77.5% (95% CI: 76.5, 78.5%) by age 65‐74. Population attributable fractions of dementia increased from 22.7% (95% CI: 15.3, 30.1%) for vascular risk factors measured at age 45‐54 to 45.4% (95% CI: 32.5, 58.3%) for vascular risk factors assessed at age 65‐74 (see Figure). Attributable fractions were larger in females, participants without apolipoprotein ε4 alleles, and Black participants (Figure).

**Conclusion:**

Between 23% to 45% of incident dementia cases by age 80 were attributed to a combination of hypertension, diabetes, or current smoking up through age 74. Attributable fractions for the combined impact of vascular risk varied by key demographics. Public health strategies targeting modifiable vascular risk factors could reduce dementia risk and increase healthy lifespan.